# Conformational trapping of an ABC transporter in polymer lipid nanoparticles

**DOI:** 10.1042/BCJ20210312

**Published:** 2022-01-20

**Authors:** Naomi L. Pollock, James Lloyd, Carlotta Montinaro, Megha Rai, Timothy R. Dafforn

**Affiliations:** 1 School of Biosciences, University of Birmingham, Birmingham, U.K.; 2 Department of Biological Sciences, University of Southampton, Southampton, U.K.

**Keywords:** ABC transport proteins, conformational trapping, lipid nanoparticles, membrane proteins

## Abstract

ATP-binding cassette (ABC) proteins play important roles in cells as importers and exporters but as membrane proteins they are subject to well-known challenges of isolating pure and stable samples for study. One solution to this problem is to use styrene-maleic acid lipid particles (SMALPs). Styrene-maleic acid (SMA) can be added directly to membranes, forming stable nanoparticles incorporating membrane proteins and lipids. Here we use Sav1866, a well-characterised bacterial protein, as a proxy for ABC proteins in general. We show that stable and monodispersed Sav1866 can be purified at high yield using SMA. This protein can be used for biophysical characterisations showing that its overall structure is consistent with existing evidence. However, like other ABC proteins in SMALPs it does not hydrolyse ATP. The lack of ATPase activity in ABC–SMALPs may result from conformational trapping of the proteins in SMALPs. Undertaken in a controlled manner, conformational trapping is a useful tool to stabilise protein samples into a single conformation for structural studies. Due to their inability to hydrolyse ATP, the conformation of Sav1866–SMALPs cannot be altered using ATP and vanadate after purification. To achieve controlled trapping of Sav1866–SMALPs we show that Sav1866 in crude membranes can be incubated with ATP, magnesium and sodium orthovanadate. Subsequent solubilisation and purification with SMA produces a sample of Sav1866–SMALPs with enhanced stability, and in a single conformational state. This method may be generally applicable to vanadate-sensitive ABC proteins and overcomes a limitation of the SMALP system for the study of this protein family.

## Introduction

ATP-binding cassette (ABC) proteins undertake fundamental biochemical processes: uptake of nutrients into cells, and efflux of toxins out of them. In some cases, these proteins display immense polyspecificity and are able to bind and transport hundreds of structurally diverse substrates [1]. In humans, the activity of ABC proteins is influential in common diseases like cancer as well as rare genetic diseases including cystic fibrosis, Tangier disease and Stargardt syndrome. In plants and bacteria, ABC proteins direct nutrient acquisition, germination of seeds [2] and protection against xenobiotics [3]. As a result, the study of these proteins is a priority across many bioscience disciplines.

The typical architecture of an ABC protein consists of twelve transmembrane (TM) helices and two nucleotide-binding domains (NBDs). These may be encoded by one or several genes [3,4], and domains may be arranged as pseudo-symmetrical monomers (e.g. P-glycoprotein and CFTR), homodimers (BmrA, Sav1866) or heterotetramers (MalFGK_2_). Movement of substrates is powered by the hydrolysis of ATP at the NBDs, which dimerise in response to ATP binding [5]. Either the binding event itself, or ATP hydrolysis, powers conformational rearrangement in the TM region and movement of the transport substrate across the membrane. Unravelling the details of this process and the molecular basis of polyspecific substrate binding and resulting multidrug resistance issues is an ongoing research problem. This pursuit has often been frustrated by the low yields and instability of these proteins when attempts are made to extract them using conventional approaches involving detergent.

One emerging solution to this problem is the use of styrene-maleic acid lipid nanoparticles (SMALPs) to extract and stabilise ABC proteins [6]. SMALPs are increasingly recognised as a useful alternative to traditional detergents. They are formed when styrene-maleic acid (SMA) is added directly to crude membrane samples, disrupting the bilayer continuum by spontaneously forming nanoparticles that include membrane proteins along with their local lipid environment [7]. There is mounting evidence that the preservation of this lipid environment promotes both the functional and structural stability of many target proteins [8–10]. A simple example of this is the fact that experiments with SMA can be carried out at room temperature without the loss of structural or functional properties of the protein [6,9,11]; this is considered unusual for solubilised membrane proteins. In the last decade, the technology has been used successfully for isolation of ATP-binding cassette (ABC) proteins including P-glycoprotein, CFTR and MRP1 [6] but despite these successes there are some technical constraints that prevent the application of the SMALP technology to all proteins.

One such limitation of the SMALP method is the sensitivity of the SMA polymer to divalent cations [7,12,13], with the onset of SMALP destabilisation occurring at 2 mM Mg^2+^ [6,14]. For ABC proteins, magnesium at millimolar concentrations is an essential co-factor in the efficient binding and hydrolysis of ATP at the nucleotide-binding domains (NBDs) of the proteins. Therefore, though it is possible to show binding of nucleotides to ABC protein in SMALP nanoparticles [6,14], there is no evidence that the ABC–SMALPs can turn over ATP at normal rates. This may be because magnesium added to protein–SMALPs is sequestered by the SMA polymer, reducing the effective magnesium concentration in solution and preventing efficient ATP hydrolysis at the NBDs. Alternatively, since ABC efflux proteins are thought to undergo extensive conformational changes during ATP hydrolysis [15], it is possible that SMALP particles physically constrain ABC efflux proteins and prevent conformational changes. The observed inhibition of ATP turnover may also be a combination of these effects.

**Figure 1.**
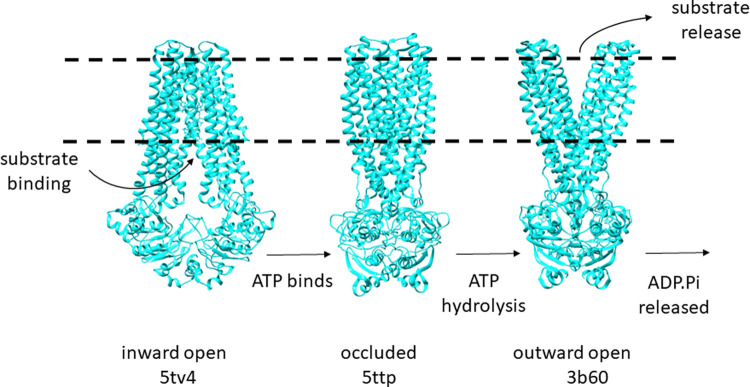
Schematic representation of the conformational cycle of a typical ABC export protein, MsbA. Different conformations of MsbA are represented in blue, with dashed black lines showing the approximate location of the membrane bilayer. The inward open conformation has high affinity for substrate(s). Binding of ATP triggers dimerisation of the NBDs as the protein enters an occluded conformation with substrate bound. ATP is hydrolysed allowing the protein to move into the outward open conformation which releases ADP and P_i_ from the NBDs. Substrate is released on the exterior of the membrane.

Recently an increased variety of amphipathic co-polymers for membrane proteins purification has been reported [11,16]. There is evidence that di-isobutylene maleic acid (DIBMA) has a higher tolerance of divalent cations, while both DIBMA and styrene maleimide (SMI) facilitate the turnover of rhodopsin where SMA does not [17]. However, SMALP-mediated constraint of the conformational motion of ABC proteins would be an advantage for some analyses, if it could be controlled. Protein samples for structural studies are often deliberately stabilised or trapped in certain conformations, and this can improve the resolution of structural information that is generated. Conformational trapping may be achieved by mutation, by addition of natural substrates or inhibitors, or by the addition of antibody fragments or nanobodies. One useful aspect of many ABC proteins is their sensitivity to orthovanadate, which exchanges for phosphate in the NBDs after ATP hydrolysis and stabilises the post-hydrolytic conformation of the protein. This leaves ADP and orthovanadate irreversibly bound in the active site and inhibits further hydrolysis of ATP [18,19] (Figure 1).

Conformational trapping of ABC proteins by orthovanadate is typically undertaken after purification of the protein. However, ABC proteins in SMALPs lack of ATPase activity, making it impossible to manipulate their conformation in this way. Therefore, here we present an alternative solution to the problem of conformational trapping of ABC–SMALPs, using orthovanadate to generate stable monodisperse samples of protein from crude *E. coli* membranes that are treated with orthovanadate prior to solubilisation with SMA.

In this study, we use the *Staphylococcus aureus* ABC transporter Sav1866 as a proxy for orthovanadate-sensitive ABC proteins in general. Sav1866 was the first ABC efflux protein to be crystallised and to yield a high-resolution structure [20]. It is orthovanadate-sensitive: the first structure as solved in its orthovanadate-trapped post-hydrolytic form. We show that Sav1866 can be purified at high yield using SMA, and that these samples are amenable to biophysical characterisation. Furthermore, we show that by altering the sequence of orthovanadate treatment and SMA-solubilisation, a conformationally monodispersed sample of the post-hydrolytic form of Sav1866 can be isolated.

## Materials and methods

DNA constructs and BL21 glycerol stocks were a gift from J.M. East (University of Southampton). SMA2000P was purchased from Cray Valley (US) and prepared for use according to the protocol described in Lee et al. [7]. Other chemicals were from Sigma–Aldrich and Fisher Scientific unless stated otherwise.

### Protein expression and isolation of cell membranes

Sav1866 was expressed in BL21 cells grown in Terrific Broth (TB: 12 g Bacto tryptone, 24 g Bacto yeast extract, 4% (v/v) glycerol, 0.17 M KH_2_PO_4_ and 0.72 M K_2_HPO_4_, 1% (w/v) glucose, 100 mg ampicillin) [21]. Glycerol stocks of BL21 cells transformed with the Sav1866 expression vector were streaked onto agar plates (1% (w/v) agar, Lysogeny broth (LB), 0.1 mg/ml ampicillin) to produce single colonies. One colony was used to inoculate fifty millilitres of broth (LB, 0.1 mg/ml Amp) which was incubated overnight at 37°C with 200 rpm shaking. Freshly autoclaved TB was inoculated with the overnight culture (50 ml per litre TB) and incubated overnight at 37°C with 200 rpm shaking until an optical density at 600 nm of 0.8 was reached. To induce expression of Sav1866, 1 mM IPTG was added and the temperature was lowered to 25°C. After overnight incubation, cells were spun down at 12 000***g*** for 20 min and the cell pellets were retained.

Cells were resuspended in ice-cold breakage buffer (50 mM Tris pH 8, 500 mM NaCl, 0.5 mM EDTA, protease inhibitors) using 10 ml buffer per 1 g cells (∼250 ml total). Resuspended cells were passed four times through the C3 lysis device (chilled to 4°C) at 1000 psi pressure. Unbroken cells and organelles were removed from the lysate by centrifugation at 12 000***g*** for 20 min (4°C). The supernatant was retained and crude membranes were isolated from it by centrifugation at 100 000***g*** for 45 min. The resulting pellets were weighed and the membranes were resuspended to 60 mg wet weight per millilitre of buffer (20 mM Tris pH 8, 150 mM NaCl).

### Vanadate treatment of crude membranes

Crude *E. coli* membranes at 60 mg/ml wet weight were incubated at room temperature for 60 min with 5 mM vanadate, 5 mM ATP and 2.5 mM MgCl_2_. Control samples were incubated under the same conditions with ATP and MgCl_2_, and without any additives. These samples were taken forward for solubilisation according to the standard protocol.

### Solubilisation and purification of Sav1866

To solubilise Sav1866 in SMA, crude *E. coli* membranes at 60 mg/ml were mixed with an equal volume of solubilisation buffer (20 mM Tris, 150 mM NaCl, 5% (w/v) SMA) to give a final concentration of 2.5% SMA and 30 mg/ml membranes. For DDM solubilisation, the same conditions were used but the SMA was replaced with 2% (w/v) dodecylmaltoside (DDM). The solubilisation mixture was incubated on a rocking platform for 90 min at room temperature for SMA solubilisation or 4°C for DDM solubilisation. Insoluble material was removed by centrifugation (100 000***g***, 45 min, 4°C) and the soluble material was taken forward for purification.

The SMA-soluble material was incubated with NiNTA resin (Generon, U.K.) overnight at 4°C using 1 ml resin per 25 ml soluble material. The NiNTA resin/soluble fraction mixture was transferred to a gravity column (Generon, U.K.) and unbound material was collected. To purify Sav1866–SMALPs the column was washed with 10 column volumes (CV) each of buffer A (20 mM Tris pH 8, 150 mM NaCl), buffer B (buffer A + 10 mM imidazole) and buffer C (buffer A + 20 mM imidazole). Each wash was collected as a single fraction (labelled W0, W10 and W20). Pure Sav1866–SMALPs were eluted from the column as twenty 1 ml fractions using buffer D (20 mM Tris pH 8, 150 mM NaCl, 500 mM imidazole) labelled E1-20.

The purification of Sav1866 in DDM (Anatrace, U.K.) was as described in the previous paragraph, with a few modifications. The DDM-soluble material was incubated with NiNTA resin for 2 h at 4°C. The purification buffers were supplemented with 0.05% (w/v) DDM and the final wash step (W40) used 10 CV buffer A supplemented with 0.05% (w/v) DDM and 40 mM imidazole. For both purifications, selected fractions were analysed by SDS–PAGE (12% acrylamide gels) and staining with InstantBlue stain (Expedeon Ltd, U.K.).

Sav1866–SMALPs were purified further by size exclusion chromatography (SEC). Fractions from the affinity purification containing Sav1866 were pooled and exchanged into buffer A (20 mM Tris pH 8, 150 mM NaCl) using a PD10 column (Cytiva, U.K.). The sample was concentrated to ∼250 µl and resolved on a Superdex 200 Increase 10/300 column (Cytiva, U.K.) run at room temperature 1 ml/min and collected in 0.5 ml fractions. The same protocol was used for SEC of Sav1866 in DDM using buffer A supplemented with 0.05% (w/v) DDM.

Selected fractions from NiNTA and SEC chromatography separations were analysed using SDS–PAGE and native SMA–PAGE using 4–20% gradient Tris-glycine gels and the Laemmli buffer system, with SDS omitted for the native SMA–PAGE protocol as previously described [22]. Gels were developed with protein stain, or transferred to nitrocellulose membrane for immunoblotting with an anti-6His antibody. A secondary horseradish peroxidase (HRP) antibody was used to develop the immunoblots with enhanced chemiluminescent (ECL) reagents (Cytiva, U.K.).

### Functional analysis of crude membranes and purified protein

Hydrolysis of ATP was measured using a modified Chifflet assay to quantify liberated phosphate [23,24] and phosphate standards were used to enable quantification of the phosphate signal. Crude *E. coli* membranes with and without Sav1866 expression, and purified Sav1866–SMALPs, were tested. The amount of membrane added to the assay was normalised by diluting both samples to an optical density at 600 nm of 1. Samples of membranes and pure protein were incubated with 0–2 mM ATP for 20 min at 37°C. Inhibition by orthovanadate was tested by the addition of 5 mM Na_3_VO_4_ (V_i_) prior to the addition of ATP.

Inhibition of ATPase activity by SMA was tested in two ways. First, 0.1% (w/v) SMA was added to membranes with and without induction of Sav1866 expression prior to the addition of Na_2_ATP (0.25–2 mM) to the samples. In addition, the maximal ATPase activity of membranes was tested with 2 mM Na_2_ATP at a range of SMA concentrations (0.0125–2.5% w/v). Control samples were prepared including 2 mM Na_2_ATP and no SMA, and background signal of ATP and SMA alone was subtracted from the test samples (induced and uninduced membranes).

In all cases, the reaction was stopped after 20 min by the addition of 10% SDS. The liberation of inorganic phosphate from the ATP was detected using Chifflet reagents, which produced a blue colour detectable by absorbance at 560 nm.

### Circular dichroism spectroscopy of pure protein

The secondary structure of Sav1866 in different conformational states was determined using circular dichroism (CD) spectroscopy (Jasco J1500-150 instrument). Using a centrifugal concentrator, Sav1866–SMALPs were exchanged into a buffer of 20 mM sodium phosphate pH 8 and 20 mM NaF to minimise absorbance in the far-UV range. Sav1866 in DDM was analysed using CD spectroscopy in order to compare its stability to Sav1866–SMALPs. Sav1866 was exchanged into a CD buffer (20 mM sodium phosphate pH 8, 20 mM NaF, 0.05% (w/v) DDM).

Protein samples (0.15 mg/ml, 0.5 ml) were transferred to a 1 mm pathlength quartz cuvette. The CD signal was measured for samples with and without orthovanadate treatment at either 15°C, or at incremental temperatures from 15–90°C. Flow-through from the concentrator used to prepare each sample was used as the buffer blank and this signal was subtracted from the signal for samples containing Sav1866–SMALPs.

### Limited proteolysis of Sav1866

Limited proteolysis was used to compare the extent of proteolytic degradation of Sav1866 in the presence of trypsin. Briefly, pure samples of Sav1866–SMALP, with and without orthovanadate treatment, were incubated on ice. After the addition of trypsin at 5 µg/ml, aliquots of protein were withdrawn at the indicated time points (1, 2, 5, 10, 20 and 60 min) and immediately added to Laemmli sample buffer (LSB) at 70°C to quench the proteolytic reaction. The samples were resolved on 12% Tris-glycine SDS–PAGE gels and stained with Instant Blue stain.

### Small-angle X-ray scattering

Samples of Sav1866–SMALPs were submitted for SAXS analysis to B21 at Diamond Light Source (Oxfordshire, U.K.). The beamsize is <75 µm, camera length 4.014 meters and the resolution range in 0.0031–0.38 Å^−1^. Scattering was recorded at four dilutions, but only the undiluted sample was judged suitable for analysis. Signal from the buffer (20 mM Tris pH 8, 150 mM NaCl) was automatically subtracted from the protein signal and the resulting traces were analysed in the ATSAS 3.0 [25] and ScÅtter [26] software suites. The radius of gyration (R_g_) and scattering intensity at q = 0 (I[0]) were calculated using Guinier analysis, and distance distribution plots. Kratky plots were prepared to examine the degree of folding of the samples. Finally, low resolution structures of Sav1866 were calculated using the DAMMIF programme. The analysis parameters are listed in (Table 1) [27].

**Table 1:** **Details of SAXS experiments and analysis parameters** [27]

**(a) Sample details**	
Organism	*Staphylococcus aureus*
Source	*Escherichia coli* BL21
Description — sequence (including tags) + bound ligands/modifications, etc.	Sav1866-10His
Extinction coefficient	n.d.
M from chemical composition	64 863 Da
Solvent details	20 mM Tris pH 8, 150 mM NaCl
**(b) Structural parameters**	
Guinier analysis	
I(0)	0.038
R_g_	55.8
qR_g_ range	0.66–1.30
Quality/aggregation (from autorg)	96%/2.6%
P(r) analysis	Sav1866
I(0)	0.036
R_g_	50.5
d_max_	145
q range	0–0.143
Quality-of-fit parameter (autoGNOM output file)	0.8328
V_P_ and/or V_c_	589423 A3
**(c) Shape model-fitting**	
q range for fitting	0–0.143
Symmetry/anisotropy assumptions	P1 and P2; none
Model precision (DAMMIF log result)	0.0170

### Data plotting and analysis

Graphs were plotted in GraphPad Prism 9. The mean and 95% confidence interval of the amount of phosphate detected in each sample from the ATPase assay was displayed (*n* = 3). A Michaelis–Menten curve fit was used in GraphPad to determine the maximal ATPase activity (V_max_) of samples after ATPase assays. Statistical analysis was also undertaken in GraphPad using repeated measures one-way analysis of variance (ANOVA) and Bonferroni's post-hoc multiple comparison tests. Differences between samples were regarded as significant where *P* < 0.05.

Chimera and ChimeraX [28] were used to display PDB files of protein structures (Sav1866: 2HYD; McjD: 5OFR ADP.V_i_-bound; 5OFP inward apo) and models built from SAXS data analysis. The buried solvent accessible surface area (SASA) of McjD in its open apo and closed ADP.V_i_-bound conformations [29] were calculated using the interface command in ChimeraX. The ‘molmap' command was used in Chimera to generate molecular envelopes from the dummy atom models at 20 Å resolution. The ‘fit in map' command was used to quantify the fit of Sav1866 crystal structure (2hyd) into the molecular envelopes.

## Results

### Sav1866 can be purified in SMALPs

**Figure 2.**
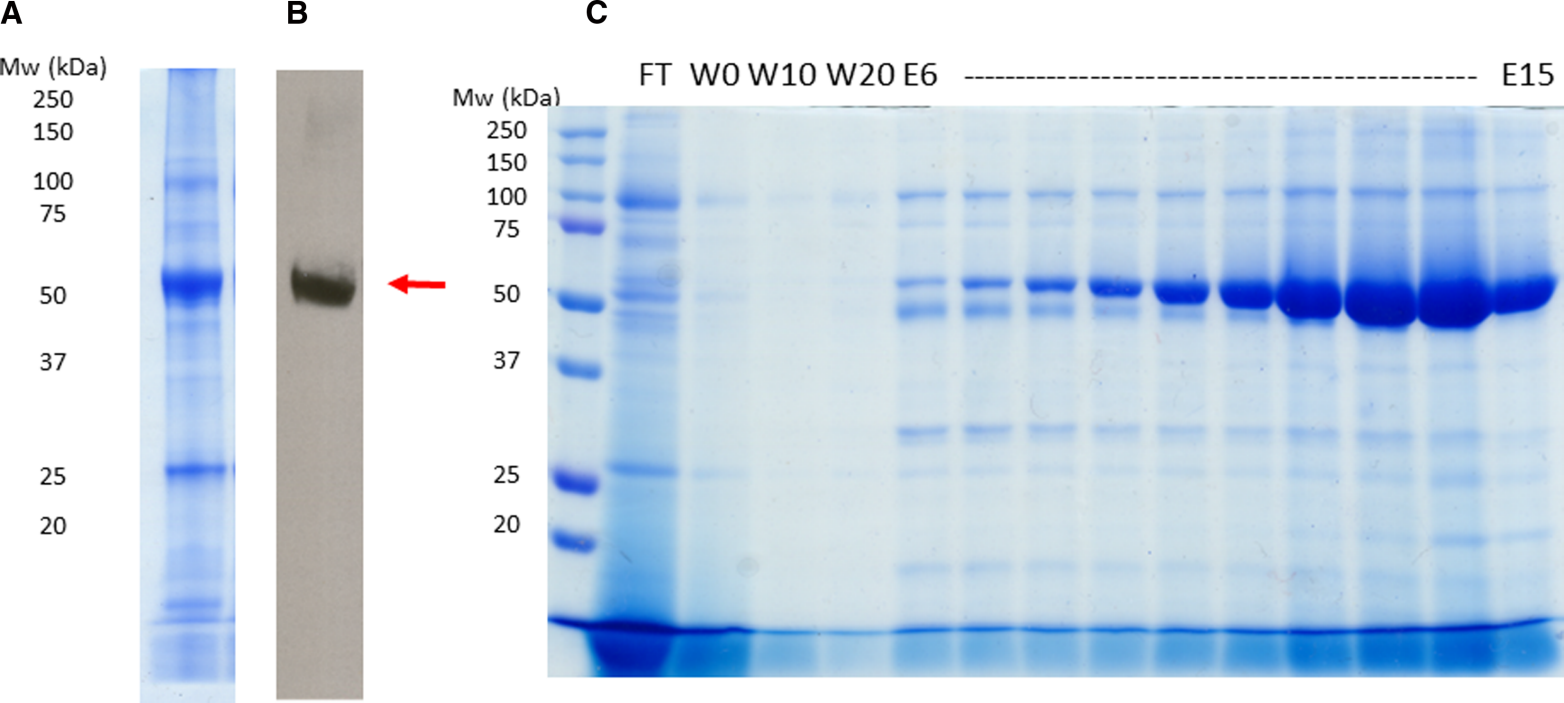
Expression and purification of Sav1866 analysed by SDS–PAGE and immunoblotting. Poly His tagged Sav1866 was detected in crude *E. coli* membrane using SDS–PAGE followed by Coomassie staining (**A**) and immunoblotting (**B**). Membranes were solubilised with 2.5% SMA and Sav1866 SMALPs were isolated using Ni-affinity purification. The SDS–PAGE (**C**) shows the flow through fraction (FT); 0, 10 and 20 mM imidazole washes (W0, W10, W20) and the elution fractions from 6 15 ml (E6 15) detected with Coomassie stain.

**Figure 3.**
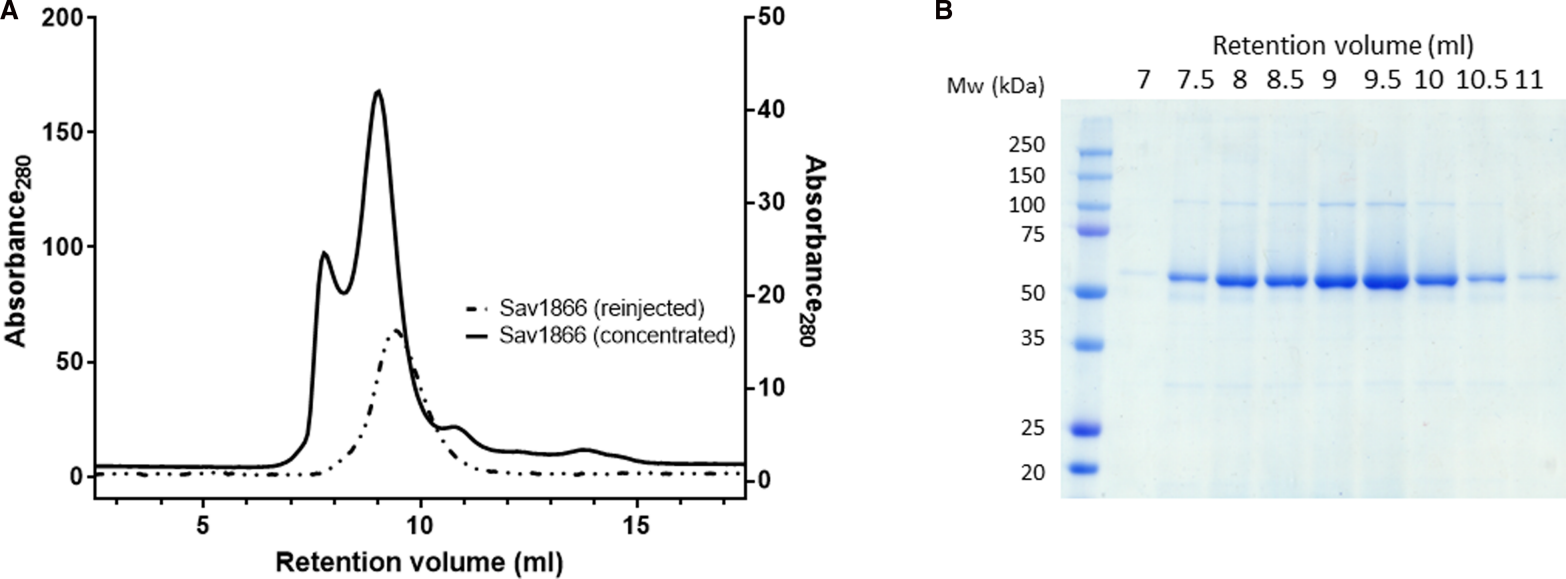
Purity of Sav1866–SMALPs after size-exclusion chromatography (SEC). A size-exclusion column was used as a second purification step for Sav1866–SMALPs (**A**). The sample was concentrated after affinity purification and injected onto the column (solid line, left hand axis). A sample from the peak fraction was reinjected (dashed line, right hand axis) to assess its monodispersity. A 10 µl sample of each fraction from the initial SEC experiment was analysed using SDS–PAGE and Coomassie staining (**B**).

Sav1866 was overexpressed in *E. coli* cells showing a dominant band at 65 kDa in the crude membrane fraction after SDS–PAGE and Coomassie staining (Figure 2A). The identity of the 65 kDa band was confirmed using immunoblotting against the poly-Histidine tag (Figure 2B). Affinity purification using an N-terminal 10-His tag yielded up to 10 mg Sav1866–SMALPs per litre of cultured cells at high purity (Figure 2C and Supplementary Figure S1A). Size exclusion chromatography was used as a second purification step (Figure 3A). It gave a minor increase in the purity of Sav1866–SMALPs (Figures 2C, 3B) but more importantly removed aggregated protein and allowed different oligomeric assemblies of the protein to be separated (Supplementary Figure S1B).

Size-exclusion chromatography was also used to assess the monodispersity of Sav1866–SMALPs. Fractions eluted from the NiNTA affinity resin were exchanged into imidazole-free buffer to reduce aggregation of the Sav1866–SMALPs during subsequent concentration in a centrifugal concentrator [30]. Despite the buffer exchange step, most samples of Sav1866–SMALPs contained some aggregated particles, which eluted in the void volume of the SEC column (Figure 3A, solid line). Nonetheless, it was possible to isolate monodisperse Sav1866 by carefully selecting the fractions to take forward after SEC: a repeated SEC analysis of protein that eluted from the column at 12 ml showed a single peak, which is indicative of monodisperse protein (Figure 3A, dashed line). Therefore, the Sav1866–SMALPs used for CD and SAXS analyses (see 1.3.2) were taken from this fraction of the first SEC purification. After the two-step purification (NiNTA and one SEC) the yield of Sav1866–SMALPs was up to 10 mg per litre of cultured cells. Furthermore, these samples of Sav1866 were concentrated in a centrifugal concentrator to ∼100 mg/ml (Supplementary Figure S1A) without any evidence of precipitation of the protein. Sav1866 purified in the non-ionic detergent DDM as a comparison with the Sav1866–SMALPs gave a similar purity (Supplementary Figure S2A,B).

### Sav1866–SMALPs are stable and monodisperse

**Figure 4.**
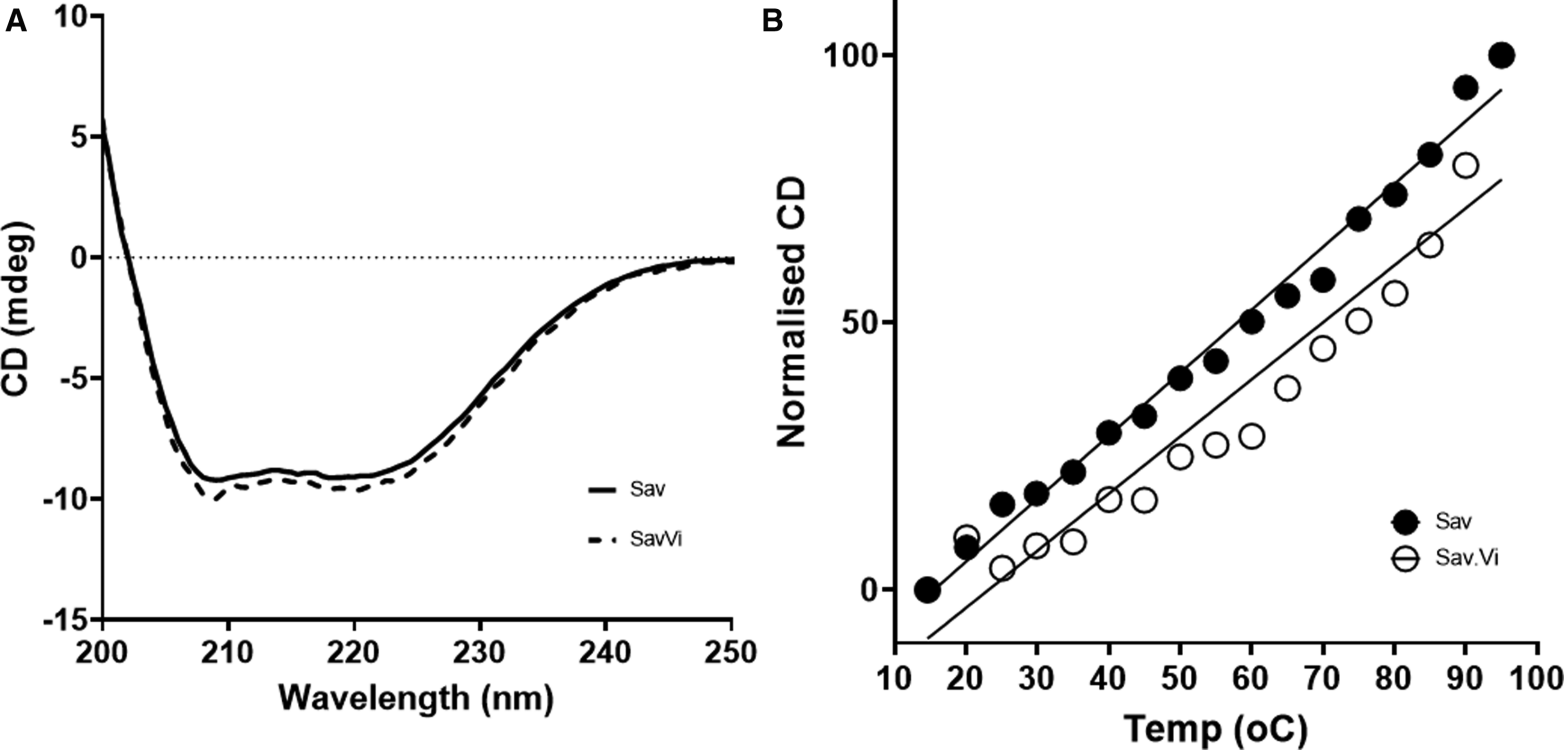
Circular dichroism spectroscopy of Sav1866–SMALPs with and without vanadate trapping. Spectra of Sav1866 (Sav) and Sav1866 in complex with vanadate (Sav.Vi) were recorded at 15°C (**A**). To compare their thermal stability, the CD signal at 220 nm from was monitored during a temperature increase from 15–90°C. The CD signal at 220 nm was normalised and plotted against the temperature (**B**).

Circular dichroism spectroscopy was used to examine the secondary structure of Sav1866. The CD signal was dominated by alpha helical signatures, apparent from the strong negative signal at 208 nm and 222 nm (Figure 4A, solid line). The CD signal at 15°C was almost indistinguishable between Sav1866–SMALPs and Sav1866 solubilised in DDM (Supplementary Figure S2C). This suggested that the secondary structure of the protein was very similar in the two solubilisation systems (Supplementary Figure S2C).

When Sav1866–SMALP samples were subjected to a temperature increase from 15°C to 90°C a change in the CD signal at 222 nm was observed (a decrease in the negative signal). This was plotted as the percentage change in the signal at that wavelength against the temperature (Figure 4B, black circles). The data indicated that some thermally induced unfolding of the protein occurred. However, there was no evidence of a co-operative unfolding event, which is typically observed in temperature-induced unfolding of soluble proteins. Thermal unfolding of Sav1866 in DDM showed a similar trend to the SMALP-solubilised material (Supplementary Figure S2C).

To examine the structure of the SMALP-solubilised Sav1866 in more detail a small-angle X-ray scattering (SAXS) analysis of Sav1866–SMALPs was carried out. SAXS experiments have been used previously to examine the structure of proteins in SMALPs [31]. These data provide important information on the morphological dispersity of the particles in solution as well as some low resolution data on their structures.

**Figure 5.**
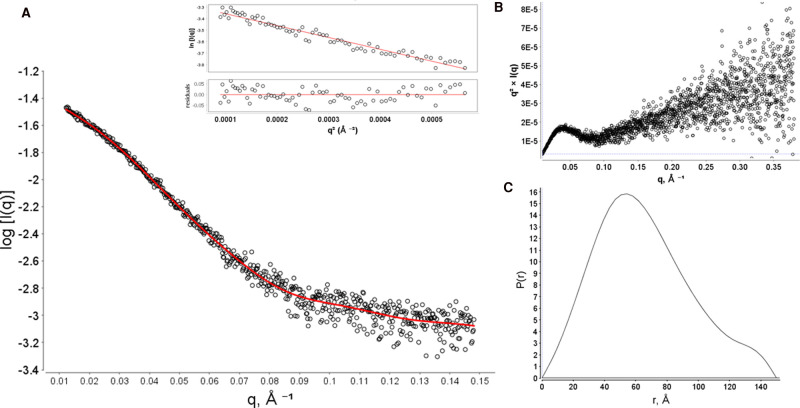
Small angle X-ray scattering analysis of Sav186–SMALPs. SAXS data for Sav1866 SMALPs was plotted as a log_10_ plot (**A**) and a Guinier plot to determine the radius of gyration (inset). Other aspects of the sample were examined by transforming the data into the Kratky (**B**) and distance distribution (**C**) plots.

The SAXS data on Sav1866–SMALPs showed that the samples had minimal aggregation, inferred from the linearity of the Guinier plot of low values of *q*^2^ (Figure 5A, inset). The maximum dimension (D_max_) of the protein was 145 Å, similar to the known structure of Sav1866, which is ∼120 Å in its longest dimension (Figure 5B). A Kratky analysis of the Sav1866–SMALPs showed that the signal at q*R_g _> 4 did not return to 0, as is expected for folded proteins (Figure 5C). However, due to the limited SAXS data for proteins in SMALPs it is difficult to draw conclusions from this observation, and CD spectroscopy showed that the Sav1866–SMALP sample had the expected secondary structural fold. More extensive studies of protein–SMALPs by SAXS may be needed to understand this better.

**Figure 6.**
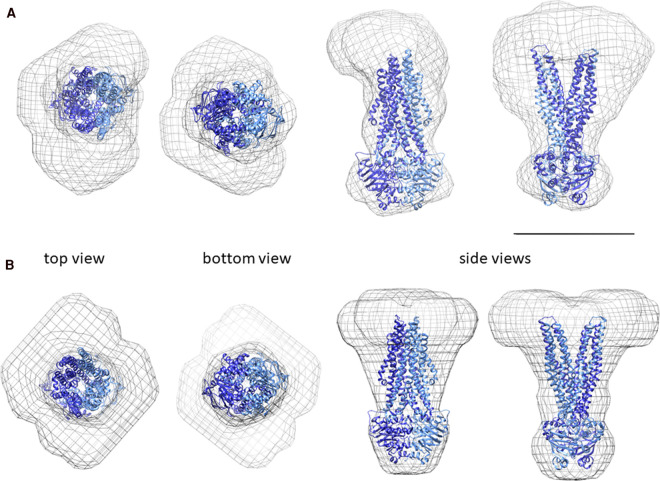
Structures of Sav1866–SMALPs from SAXS analysis. Models of Sav1866–SMALPS at 20 Å resolution were generated from SAXS data using p1 (**A**) and p2 (**B**) symmetry. The models are fitted with the 2HYD structure of Sav1866 (blue/cyan). In this representation, the NBDs are the ‘bottom' and the cytoplasmic side the ‘top' of the protein. The scale bar (black line) is 100 Å in length.

This availability of good-quality data for the material allowed models of Sav1866 to be built from the SAXS data. Models were produced with both p1 and p2 symmetry applied to the data. This considered the homodimeric arrangement of Sav1866, but also allowed for possible asymmetry in the arrangement of the protein and SMALP disc. In both cases, the X-ray structure of Sav1866 (2hyd) was fitted into the calculated molecular envelopes (Figure 6). This further confirmed that the protein assembled as monodisperse dimers in SMALPs.

Both SAXS structures showed density that could be attributed to the protein and to the SMALP, the latter forming a disc-like density at one end of the particle. These structures suggest that Sav1866 was embedded within the lipid bilayer region of the SMALP. The NBDs and cytoplasmic helices of the known structure of Sav1866 fitted well into both models, with the symmetrical (p2) model showing a particularly good fit in this region.

### Sav1866 is active in crude membranes but inactive in SMALPs

**Figure 7.**
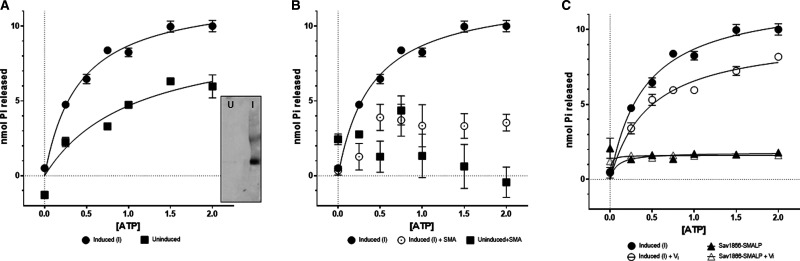
Effect of vanadate and styrene-maleic acid on the ATPase activity of Sav1866. The hydrolysis of ATP was measured in crude membranes with and without the induction of Sav1866 expression (**A**). The inset immunoblot shows the presence of Sav1866 in the induced (I) membranes and its absence in the uninduced (U) membranes. Inhibition of ATPase activity by SMA was measured in crude membranes in the presence and absence of 0.1% SMA (**B**). In each case the plots show the mean and standard deviation of the data (*n* = 3) with Michaelis–Menten non-linear fit, where possible. The effect of 5 mM vanadate (+Vi) treatment was determined in membranes (C, open circles) and pure Sav1866–SMALPs (C, open triangles).

There are several reports that purified ABC proteins in SMALPs lack measurable ATPase activity [6]. We determined that Sav1866 was active in crude membranes, and tested the effect of SMA on its ATPase activity. Crude membranes from *E. coli* carrying the pet-19b:*sav1866* plasmid but without expression of the plasmid (uninduced) were compared with those with Sav1866 overexpressed (induced) (Figure 7A). An immunoblot of the different membrane samples showed that no Sav1866 was expressed in the uninduced membranes, meaning that ATPase activity in these samples could be regarded as the background ATPase activity of BL21 membranes. Purified Sav1866–SMALPs were also analysed. The maximal ATPase activity (V_max_) of these samples, expressed in terms of the amount of phosphate released from ATP over the duration of the assay, are presented in Table 2.

**Table 2:** Maximal ATPase activity of crude membranes and pure Sav1866–SMALPs

Sample name	V_max_ (nmol P_i_ released)		
	Sample	Sample + V_i_	Sample + SMA
Uninduced membranes	9.7	N.D.	1.4
Induced membranes	12.2	9.6	4.2
Pure Sav1866-SMALPs	1.8	1.6	N/A

V_max_ values were derived using Michaelis–Menten kinetic fitting of the data. For some samples the V_max_ could not be determined (N.D.)

Crude membrane samples were used in order to determine whether Sav1866 was active prior to solubilisation with SMA. Due to the presence of many ATPase proteins in *E. coli* membranes, the uninduced membranes showed turnover of ATP (Figure 7A, squares). The hydrolysis of ATP was significantly faster in membranes containing Sav1866 (Figure 7A, circles) than those without it (*P* < 0.05, Tables 2 and 3). This indicated that much of the measured ATP hydrolysis by induced membranes could be attributed to the activity of Sav1866 rather than other ATP-hydrolysing proteins that were also present in the crude membrane fractions.

Sav1866–SMALPs displayed very little turnover of ATP (Figure 7C, triangles; Table 2). This showed that despite its ability to hydrolyse ATP in the crude membranes, after encapsulation in SMALPs, Sav1866 no longer functioned as an ATPase (Figure 7 and Table 2).

### SMA inhibits the total ATPase activity of crude membranes

To understand more about the effect of SMA on ATPase activity, we examined its effect in crude membranes at a range of SMA concentrations, both with and without Sav1866 expressed. Addition of 0.1% (w/v) SMA to both induced and uninduced membranes significantly reduced the turnover of ATP by the samples compared with the rate in the absence of SMA (Figure 7B and Table 2). There was no significant difference between the induced and uninduced samples in the presence of SMA (Table 3), indicating that the inhibition reached both Sav1866 and other ATPase proteins. This differed from the orthovanadate-mediated inhibition, which had different effects on the induced and uninduced samples (1.3.5).

**Table 3:** Statistical analysis of ATPase activity assays

Sample	Comparison		*P*-value	Significant? (<0.05)
Pure protein	Effect of V_i_		>0.9999	N
	Induction of Sav1866		0.0044	Y
Crude membranes	Effect of V_i_	Induced	0.0164	Y
		Uninduced	0.3154	N
	Effect of SMA	Induced	0.0183	Y
		Uninduced	>0.9999	N

Data were compared using repeated measures one-way ANOVA followed by a Bonferroni multiple comparison test to compare different treatments of the crude membranes and Sav1866–SMALPs.

The inhibitory effect of SMA on the total ATPase activity of crude membranes was tested further using a range of SMA concentrations from 0.0125 to 2.5% (w/v). The addition of 0.0125% SMA caused a loss of 50% of the total ATPase activity of the membranes, both with and without Sav1866 expressed (Supplementary Figure S3 and Table 2). At 0.1% SMA, there was no measurable ATPase activity. Higher SMA concentrations (0.25–2.5% w/v) interfered with the Chifflet assay: blue colour appeared in the reaction mixture at these SMA concentrations in the absence of crude membranes. Regardless of this, the apparent rate of ATP turnover at higher SMA concentrations was less than half of that observed in the absence of SMA (Supplementary Figure S3).

### Orthovanadate treatment of crude membranes traps Sav1866 in a post-hydrolytic conformation

To determine the success of the orthovanadate treatment on Sav1866, and to separate it from the inhibitory effect of SMA, *E. coli* membranes with Sav1866 overexpressed were treated with sodium orthovanadate (V_i_) and ATP. Orthovanadate traps some ATPase proteins, including Sav1866, in a post-hydrolytic conformation (Figure 1) and inhibits further hydrolysis of ATP. Sav1866–SMALPs do not hydrolyse ATP so in order to produce V_i_-trapped Sav1866–SMALPs it was necessary to treat the crude membranes with V_i_ prior to solubilisation and purification of Sav1866.

To test whether the orthovanadate trapping of Sav1866 had worked, membrane samples (induced and uninduced) treated with V_i_ underwent an ATPase assay. Orthovanadate treatment reduced the ATPase activity of membranes containing Sav1866 by ∼50% (Figure 7B and Table 2). Uninduced membranes treated with V_i_ also showed a significant reduction in ATPase activity compared with the untreated samples (Figure 7B and Tables 2 and 3). However, this reduction in activity was smaller than that seen in the membranes containing a large amount of Sav1866. Given the very high level of Sav1866 expression in the crude membranes (Figure 2A,B), we considered this as proof that Sav1866 was successfully trapped in a post-hydrolytic conformation in crude membranes after orthovanadate treatment.

### Orthovanadate-trapping enhances the stability of Sav1866–SMALPs

Having used activity assays to confirm that orthovanadate was bound to Sav1866–SMALPs, we next investigated the effect of orthovanadate on the stability of Sav1866. To produce Sav1866.ADP.Vi-SMALPs, membranes underwent orthovanadate treatment and were subsequently solubilised and purified using SMA as previously described. SMA begins to precipitate in the presence of divalent cations in excess of 2 mM so the magnesium concentration was limited to 1.25 mM in the solubilisation mixture.

Circular dichroism spectroscopy was used to compare the secondary structure of Sav1866 purified from membranes with and without orthovanadate treatment. These were indistinguishable from each other when analysed by CD spectroscopy (Figure 4A). This indicated that, as expected, the orthovanadate treatment did not alter the secondary structure of Sav1866. The behaviour of V_i_-treated and untreated samples of Sav1866–SMALPs in a thermal unfolding experiment was also identical (Figure 4B). As previously mentioned (1.3.2), the temperature increase from 15–90°C did not result in the type of co-operative unfolding event that is typically seen for soluble proteins. There was no evidence that orthovanadate treatment altered the thermal stability of Sav1866.

**Figure 8.**
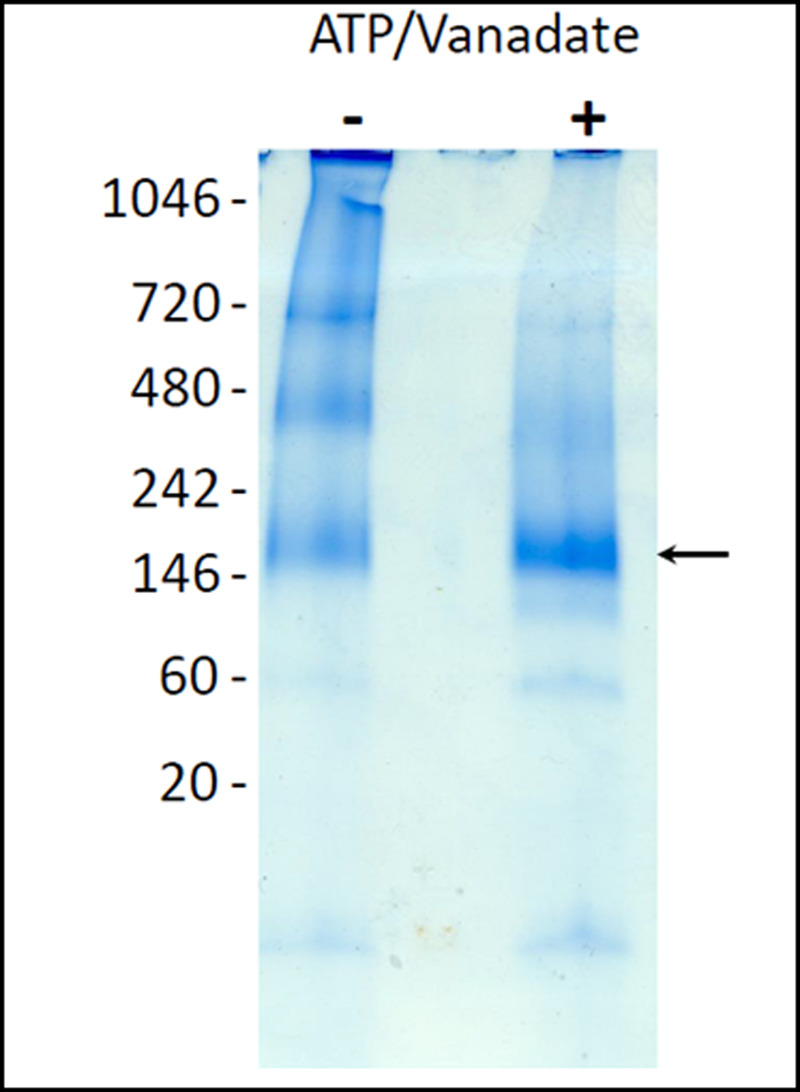
The effect of vanadate trapping on the oligomeric state of Sav1866–SMALPs. SMA–PAGE of Sav1866–SMALPs purified by affinity chromatography from crude membranes without (−) and with (+) ATP and vanadate. The migration of dimeric Sav1866 on the gel is indicated with a black arrow.

To extend the comparison of the different samples, we used native gel electrophoresis to compare the hydrodynamic properties of Sav1866 with and without V_i_. The native PAGE method, SMA–PAGE, was used to assess the oligomeric state of pure Sav1866–SMALPs prior to preparative SEC (Figure 8). By this analysis, the protein appeared to have enhanced monodispersity as a result of the conformational trapping. Samples purified from membranes treated with ATP and orthovanadate prior to SMA solubilisation showed fewer high oligomeric species compared with untreated samples (Supplementary Figure S1B). This indicated that orthovanadate-treated samples were more homogeneous than the untreated samples and that the conformation of the protein within the SMALP had been influenced by the presence of vanadate.

Finally, limited proteolysis with trypsin was used as a simple probe for the higher-order structure of Sav1866 with and without orthovanadate treatment. Due to the extensive conformational rearrangement from the pre- to post-hydrolytic conformations (Figure 1), the solvent accessibility of the protein is significantly altered. In the post-hydrolytic (V_i_-trapped) conformation, the NBDs are in contact, and it is likely that fewer trypsin-sensitive sites are available. Therefore, we hypothesised that V_i_-trapped Sav1866–SMALPs would exhibit less sensitivity to trypsin than the untrapped sample. Structures of the ABC transporter McjD have been solved in a number of different conformational states [29] which allows the compactness of each conformation to be assessed and compared with results. Using ChimeraX, the buried solvent-accessible surface areas for the nucleotide-free (‘apo') and ADP.V_i_ bound structures were calculated as 5743 Å^3^ and 7463 Å^3^, respectively. This showed that the ADP-V_i_ bound conformation was more compact, with higher buried surface area.

**Figure 9.**
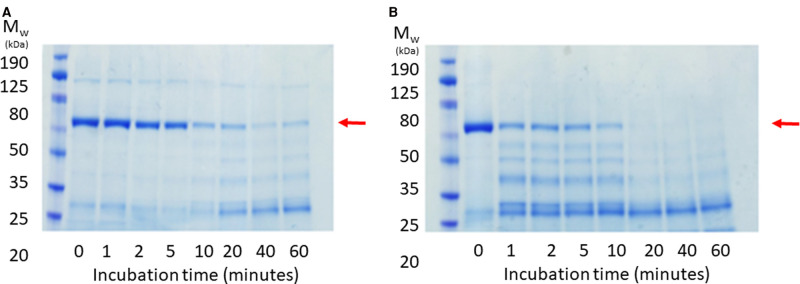
Limited proteolysis of purified Sav1866–SMALPs with and without ADP/orthovanadate. Membranes containing Sav1866 were incubated for 30 min at room temperature with ATP and MgCl_2_ in the presence (**A**) and absence (**B**) of sodium orthovanadate. Subsequent solubilisation with SMA and affinity purification yielded pure Sav1866–SMALPs, which underwent limited proteolysis with trypsin at 4°C. Samples were taken at time points between 1 and 60 min and these were analysed by SDS–PAGE and Coomassie staining to monitor the disappearance of full-length Sav1866 (indicated with a red arrow).

In the limited proteolysis experiment, the disappearance of the full-length Sav1866 band on SDS–PAGE at 65 kDa showed that Sav1866–SMALPs not treated with orthovanadate were digested by trypsin within ten minutes (Figure 9A). In contrast, Sav1866–SMALPs in complex with ADP and V_i_ were not fully digested by trypsin after 60 min had elapsed (Figure 9B). The molecular weights of the products of the digestion appeared to be consistent between the two samples, suggesting that the same trypsin sites were accessed in the two samples, but much more slowly in the V_i_-treated samples.

## Conclusions

Sav1866 was readily solubilised in SMA, and Sav1866–SMALPs could be isolated in large quantities at high purity. The Sav1866–SMALPs were amenable to analysis with circular dichroism and SAXS, but like other ABC proteins in SMALPs they did not display ATPase activity. In spite of this the secondary structure of Sav1866–SMALPs appeared similar to that of Sav1866 purified in DDM. The low-resolution structure of Sav1866–SMALPs determined by SAXS analysis showed that the purified protein was monodisperse and its overall structure agreed with the known structure of Sav1866, with the addition of density that can be attributed to the SMALP nanoparticle. Despite causing inhibition of ATPase activity, the presence of SMA did not lead to any bulk change in the secondary, tertiary or quaternary structure of the protein.

Given that ATP turnover by crude membranes can be attributed to a range of ATPase proteins, this makes it more likely that the inhibition of ATP hydrolysis seen after SMA treatment is due to the poor availability of magnesium, rather than a hindrance to conformational change. The requirement of magnesium for efficient hydrolysis of ATP is universal, whereas conformational changes are markedly different between different classes of ATPase proteins. This suggests that indiscriminate conformational trapping is not the cause of the inhibition of ATPase activity of ABC proteins in SMALPs.

Despite this observation, vanadate trapping of ABC proteins relies upon their ability to hydrolyse ATP, as it traps the post-hydrolytic conformation of the protein [32]. Vanadate treatment had no effect on the ATPase activity of purified Sav1866–SMALPs, which was close to zero both with and without the addition of orthovanadate. To be successful, the vanadate trapping could only be reliably undertaken prior to solubilisation with SMA.

We anticipated that Sav1866–SMALPs with ADP and V_i_ bound to the NBDs would exceed the stability of the untrapped samples. To test this we used both direct and indirect methods to investigate the functional and structural properties of Sav1866–SMALPs. The trapped and untrapped samples of Sav1866–SMALPs had identical spectra in CD spectroscopy (Figure 4A), indicating that their secondary structural elements were identical. However, orthovanadate-treated samples had reduced sensitivity to digestion by trypsin (Figure 9). This was consistent with the fact that the conformational cycle of ABC transporters does not appear to result in large changes to the secondary structure of the protein, but rather a rearrangement of the two halves of the transporter in relation to one another [15,29]. This rearrangement to the nucleotide-bound conformation makes ABC proteins more compact (Figure 1) compared with the nucleotide-free conformation. Therefore less surface area of the protein is exposed to solvent, reducing the availability of trypsin-sensitive cleavage sites. Native SMA–PAGE gels showed less aggregate and fewer oligomeric species of purified Vi-treated Sav1866–SMALPs. This indicates that the Vi-treatment resulted in a more monodisperse sample of the protein. Previously published data showed that the different bands observed by SMA–PAGE could be related to different oligomers of the protein observed by analytical ultracentrifugation (AUC) [22]. Considering the previous AUC data alongside the SMA–PAGE indicated that Sav1866–SMALPS with ADP.V_i_ bound were predominantly homodimers, the minimal functional unit of the protein. Overall, these data indicated that V_i_-treated Sav1866–SMALPs had enhanced stability and monodispersity compared with the untreated samples.

Ultimately, this study does not fully elucidate the mechanism of SMA-induced inhibition of ABC proteins, but provides a method to circumvent it to achieve uniform conformational trapping of the Sav1866–SMALPs. The sequence of the vanadate treatment and SMA solubilisation steps are critical. The combined effects of SMA-solubilisation and orthovanadate-trapping appear to produce a highly stable protein sample that can be produced in very large amounts. The availability of many nucleotide analogues, which capture different conformational states of ABC proteins, opens the possibility to capture ABC proteins in SMALPs in a variety of predictable conformations and is readily applicable to structural studies of ABC–SMALPs.

## Data Availability

No data in this manuscript is subject to mandatory data sharing.

## Open Access Statement

Open access for this article was enabled by the participation of University of Birmingham in an all-inclusive *Read & Publish* pilot with Portland Press and the Biochemical Society under a transformative agreement with JISC.

## Abbreviations

ABC: ATP-binding cassette
ANOVA: analysis of variance
AUC: analytical ultracentrifugation
CD: circular dichroism
CV: column volumes
DDM: dodecylmaltoside
DIBMA: di-isobutylene maleic acid
NBDs: nucleotide-binding domains
SAXS: small-angle X-ray scattering
SEC: size exclusion chromatography
SMA: styrene-maleic acid
SMALPs: styrene-maleic acid lipid particles
TB: Terrific Broth
TM: transmembrane
