# Supplementary material for: Conformational trapping of an ABC transporter in polymer lipid nanoparticles
Source: Biochem J. 2022 Jan 20;479(2):145–59. doi: 10.1042/BCJ20210312 (PMC8883494; doi:10.1042/BCJ20210312)
Supplement: Supplementary Figures 1-3 [file BCJ-479-145-s1.pdf]

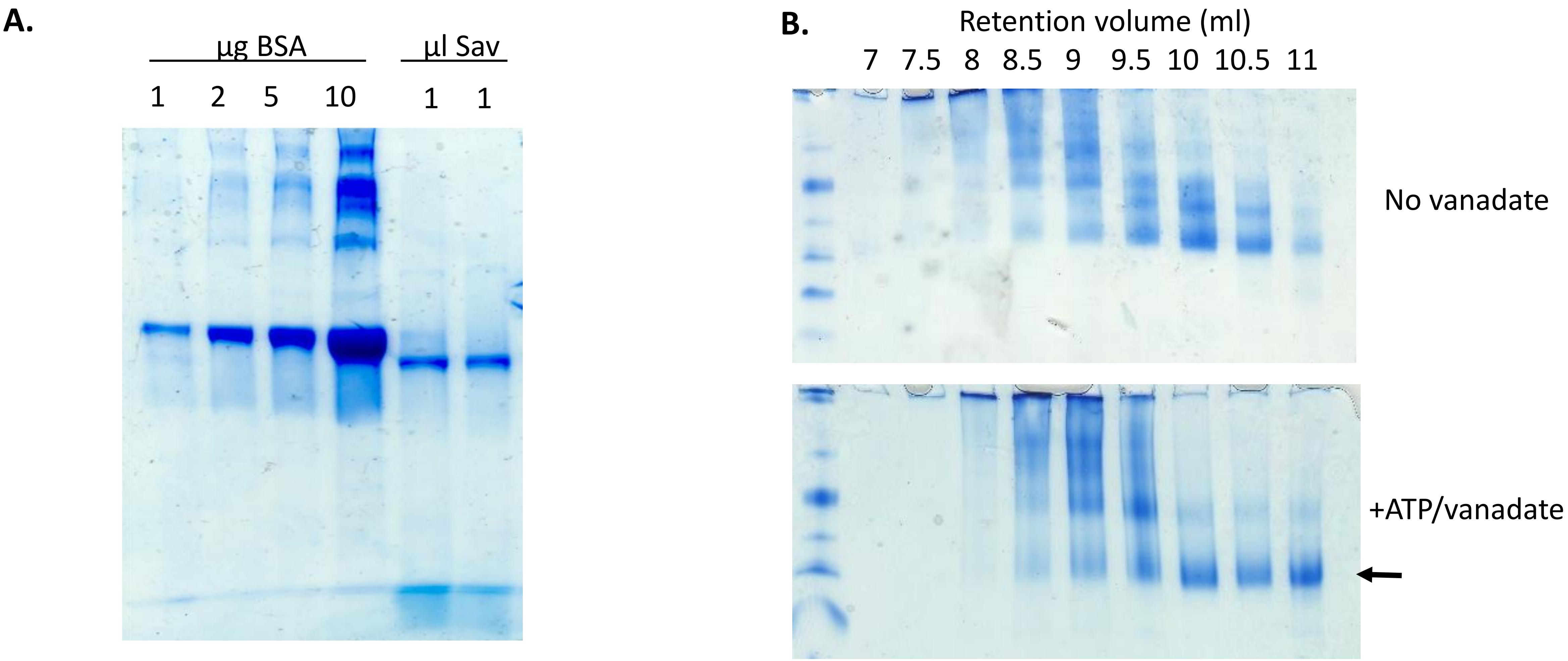

Supplementary Figure 1: Additional detail on Sav1866 purification in SMALPs. The yield of Sav1866 was estimated using a BSA standard gel (A) . The intensity of staining of 1, 2, 5 and 10 µg BSA was compared to 1 µl of Sav1866-SMALPs diluted 1:100 from the final sample. SMA-PAGE was used to assess the degree of aggregation and polydispersity in Sav1866-SMALPS after SEC with and without orthovanadate treatment (B).

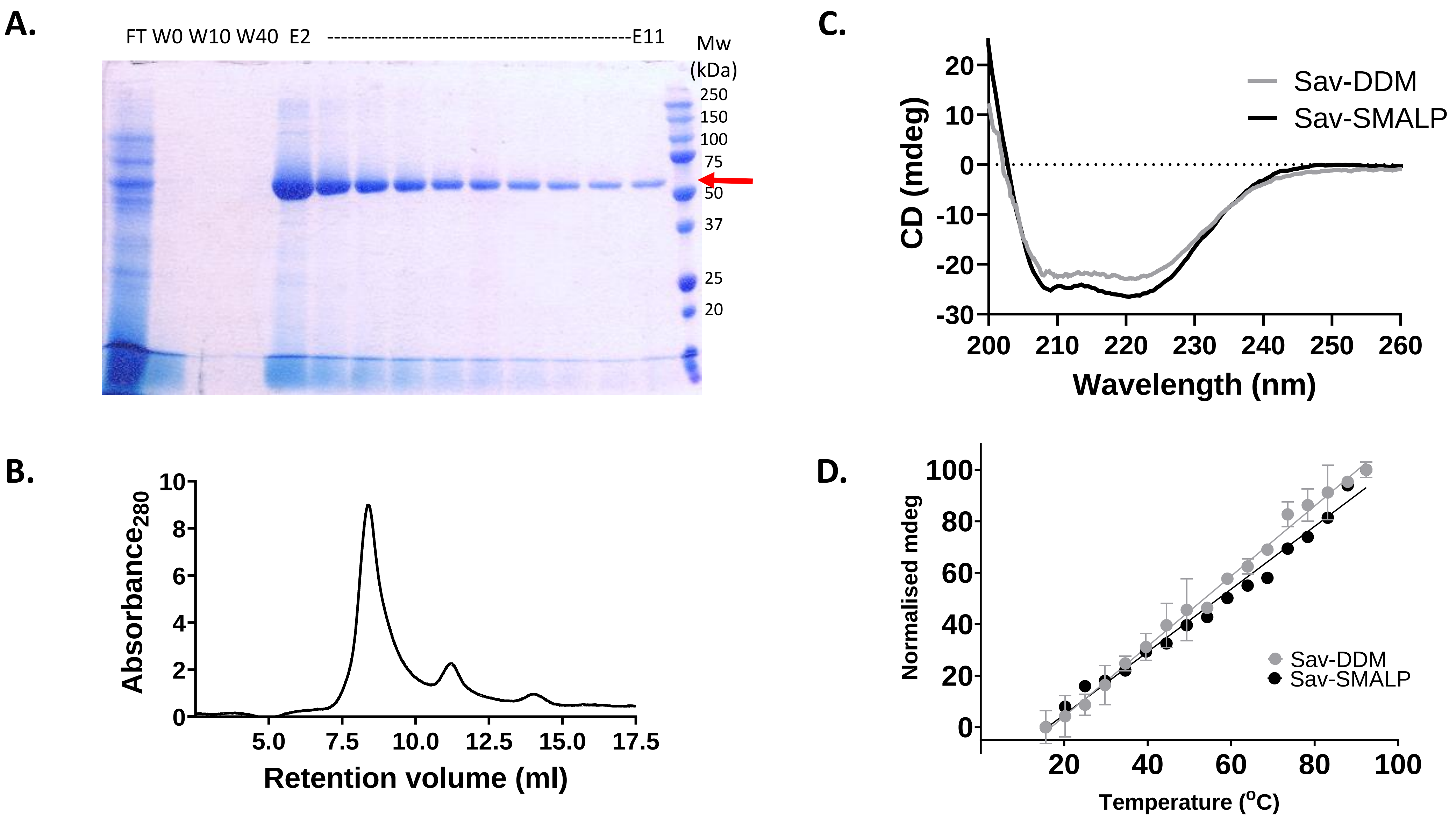

Supplementary Figure 2: Purification and characterisation of Sav1866 in DDM. Sav1866 was solubilised and purified in DDM by nickel affinity chromatography (A) and size-exclusion chromatography (B). The purified Sav1866-DDM was analysed using circular dichroism spectroscopy (grey) and compared to Sav1866-SMALPs (black) at a single temperature of 15 °C (C) and at 5 °C intervals from 15 – 90 °C monitored at 222 nm (D).

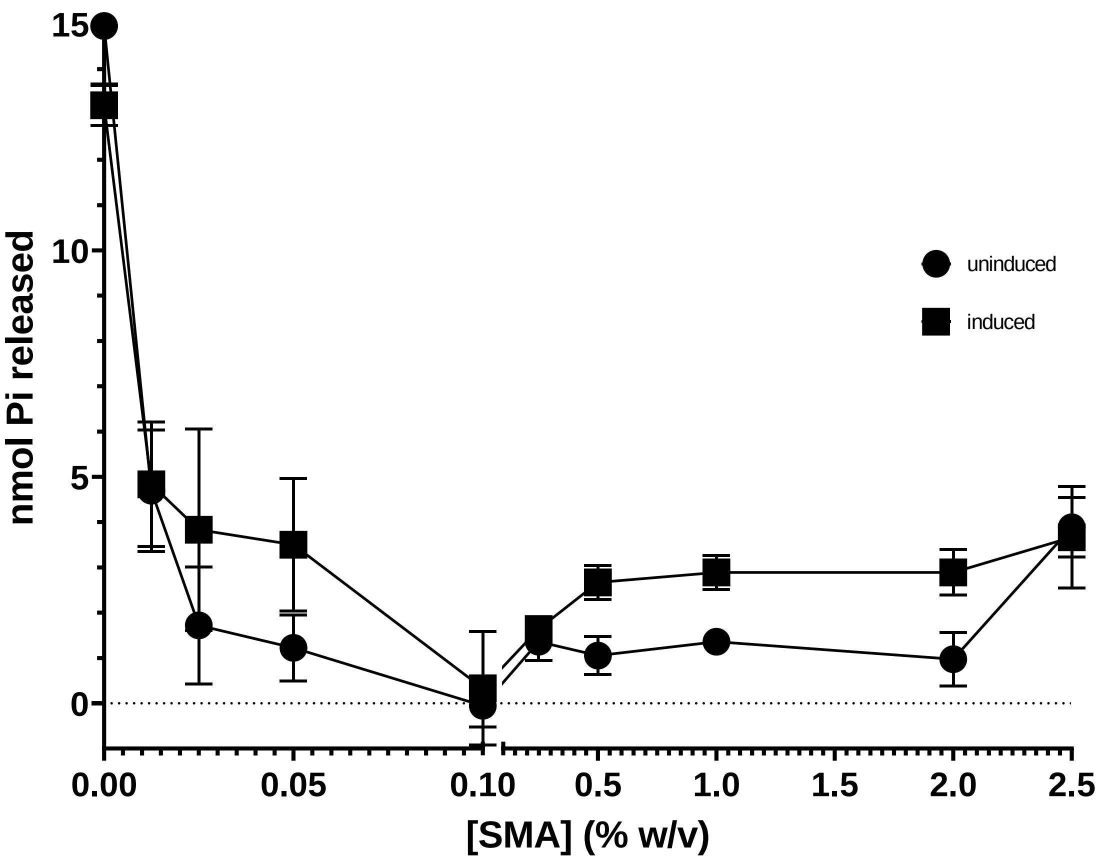

Supplementary Figure 3: Effect of SMA on the ATPase activity of crude *E. coli* membranes. Crude membranes without Sav1866 (uninduced, circles) and with Sav1866 (induced, squares) were incubated with 2 mM ATP and SMA from 0 – 2.5 % (w/v). The release of phosphate resulting from hydrolysis of ATP was quantified using the Chifflet assay.
